# Guaiacol as a Local Anæsthetic

**Published:** 1896-02

**Authors:** 


### Guaiacol as a Local Anæsthetic.

M. Championniere reports (*Gazette des Hopitaux*) a series of experiments lately made by M. Andre, a Paris physician, on the use of guaiacol as a local anæsthetic. M. Andre, as the result of an accident, received a very painful scald. It occurred to him that guaiacol, like other members of the phenol group, possessed anæsthetic properties, and he accordingly made an ointment containing it for the purpose of applying it to the burn. He was astonished at the relief of pain produced, and determined to try the effect of it subcutaneously. As a result of his experiments he affirms that it will produce effects absolutely identical with those obtained from the use of cocaine.—*Canadian Practitioner.*

